# Reflex impairment and physiology as predictors of delayed mortality in recreationally caught yellowtail snapper (*Ocyurus chrysurus*)

**DOI:** 10.1093/conphys/cox035

**Published:** 2017-06-08

**Authors:** Francesca C. Forrestal, M. Danielle McDonald, Georgianna Burress, David J. Die

**Affiliations:** 1 Cooperative Institute of Marine and Atmospheric Science, Rosenstiel School of Marine and Atmospheric Science, University of Miami, 4600 Rickenbacker Causeway, Miami, FL 33149, USA; 2 Marine Ecosystems and Society, Rosenstiel School of Marine and Atmospheric Science, University of Miami, 4600 Rickenbacker Causeway, Miami, FL 33149, USA; 3 Cape Eleuthera Institute, Rock Sound, The Bahamas

**Keywords:** yellowtail snapper, i-Stat, reflex impairment, delayed mortality, post-release survival

## Abstract

Yellowtail snapper (*Ocyurus chrysurus*) is an important part of the reef fish assemblage in the western, tropical Atlantic and is caught by both recreational and commercial fisheries in south Florida and the Bahamas. It is estimated that 80% of snapper caught within southeastern Florida waters are discarded due to minimum size restrictions. Neglecting to include information on delayed mortality of undersized fish has the potential for fishery managers to overestimate the abundance of smaller size classes and introduce bias into stock assessments. This study examines associations between reflex impairment, traditional physiological parameters and post-release mortality of undersized yellowtail snapper. Laboratory experiments exposed yellowtail snapper to a gradient, simulating capture conditions. Blood draws were obtained from a sub-sample of fish. There was a significant relationship between delayed mortality and the proportion of reflex impairment for both individual fish and groups of fish (*P* < 0.001 and *P* = 0.03). Within the sub-sample of blood-sampled fish, base excess and pH were significantly correlated to reflex impairment. Delayed mortality was significantly correlated to pH, base excess and lactate concentration. Results suggest that discarded, undersized yellowtail with more than 29% of their reflexes impaired will not survive.

## Teaser

This article estimated delayed mortality in yellowtail snapper that would be discarded in the fishery due to size regulations. Delayed mortality was estimated through reflex impairment and blood parameters associated with the stress response.

## Introduction

Yellowtail snapper (*Ocyurus chrysurus*) is an important part of the reef fish assemblage in the western, tropical Atlantic and is caught by both recreational and commercial fisheries in south Florida and the Bahamas (Johnson, 1983; Manooch and Drennon, 1987; Garcia *et al.*, 2003; Saillant *et al.*, 2012). The majority of the yellowtail snapper caught in the US come from Florida waters and this species has supported an important commercial and recreational fishery (Johnson, 1983; Ault *et al.*, 2006). To sustain this fishery, there has been a minimum size in effect for yellowtail snapper since 1983 (O’Hop *et al.*, 2012). The regulation requires that any fish caught smaller than 12 in. (30 cm) must be discarded in both the commercial and recreational fisheries. While minimum size limits aim to protect the long-term health of the stock by keeping sexually immature fish in the environment (Gabriel and Mace, 1999; Bohnsack, 2000), the stress of angling can result in delayed mortality in fish that initially survive and are released alive (Gingerich *et al.*, 2007; Campbell *et al.*, 2010; Stephen and Harris, 2010; Danylchuk *et al.*, 2014). It is estimated that 80% of snapper caught within southeastern Florida waters are discarded due to minimum size restrictions (Bartholomew and Bohnsack, 2005). To date, there have been no studies to assess the post-release mortality of discarded, undersized yellowtail snappers. Lack of information on post-release survival rates creates uncertainty in estimating the fishing mortality and the population dynamics of an economically and ecologically important reef fish population (Punt *et al.*, 2006; O’Hop *et al.*, 2012; Gilman *et al.*, 2013). Previous studies of catch and release fisheries found the majority of released fish experienced delayed mortality, rather than immediate mortality, which can bias estimates of fishing mortality (Gingerich *et al.*, 2007; Suski *et al.*, 2007).

Traditional methods of determining post-release survival of discarded fish include costly tagging experiments or measuring stress hormone concentrations in blood samples. Tagging experiments are often not logistically feasible due to economic constraints, furthermore, the tag and tagging process can act as additional stressors on the tagged fish (Diggles and Ernst, 1997; Pollock and Pine, 2007). The usefulness of blood parameters associated with the stress response to predict mortality can vary as concentrations fluctuate widely among species as well as individual fish within the same species (Davis, 2001; Raby *et al.*, 2012). Testing a holistic response to stress, such as a suite of involuntary reflexes, has been demonstrated to be an effective way to predict post-release mortality (Humborstad *et al.*, 2009; Davis, 2010; Gallagher *et al.*, 2010, 2014; Danylchuk *et al.*, 2014). The reflex action mortality predictor (RAMP) has been used by several fisheries for both teleosts and crustaceans to predict the fate of these species after being discarded from fishing activity (Davis, 2010, 2007; Raby *et al.*, 2012; Stoner, 2012).

The association of reflex impairment and mortality is a relatively new method to assess survival (Davis, 2002, 2005, 2010). The present study pairs reflex testing with measurements of traditional blood physiology parameters associated with the teleost stress response to examine reflex impairment in greater depth. Due to field conditions inherent in fishing, traditional laboratory testing is rarely feasible. Portable point of care (POC) devices, for example the i-STAT, have been gaining in popularity for use in the field in recent years as they allow for instantaneous reading of blood parameters without requiring cumbersome laboratory equipment and specially trained personnel (Gallagher *et al.*, 2010; Danylchuk *et al.*, 2014; Stoot *et al.*, 2014). These devices are configured toward clinical use in humans and other mammals and the measurements and algorithms used are not calibrated for teleost red blood cells. Differences in the size and structure of red blood cells can bias measurements taken by these potentially useful devices. Validation studies have been conducted with POC devices and traditional laboratory assays with mixed results (Harter *et al.*, 2014; Stoot *et al.*, 2014), however, with validation, these devices can provide substantial benefits in understanding physiological parameters in field settings (Gallagher *et al.*, 2010).

This study pairs several methods to assess the post-release mortality of undersized yellowtail snapper. Reflexes impaired in response to air exposure were assessed and used to predict delayed mortality. There are several stressors associated with hook and line fisheries, including fight time, hooking site, air exposure and thermal tolerances (Ferguson and Tufts, 1992; Gingerich *et al.*, 2007; Danylchuk *et al.*, 2014). While all these stresses and their interactions have important implication for survival, air exposure was used as the sole source of stress in this study as it has the most immediate management implications. In addition to reflex impairment, blood samples were taken and measured using an i-STAT and compared to reflex impairment and delayed mortality. To test the accuracy of the i-STAT, traditional laboratory assays were conducted and compared to measurements obtained with the i-STAT.

## Methods

### Collection and holding

Fish were collected from shallow water patch reefs in near shore waters off Cape Eleuthera, Eleuthera, Bahamas (24.54°N 76.12°W). The fish were caught using naturally baited light circle hooks on a rod and reel. Only fish that were mouth-hooked were retained, all others were discarded alive after de-hooking. Fish were transported back to the Cape Eleuthera Institute (CEI) and held in 3600 L flow-through tanks continuously supplied with seawater. Fish were allowed to recover and were monitored for several days. Fork length was measured (28.8 ± 3.5 cm) and all fish were tagged with Biomark^®^ 12 mm passive integrated transponder tags (Biomark^®^). Before being returned to the holding tanks and to prevent infections, tagged fish were treated by spraying Betadine on the area where the tag was inserted.

Fish were randomly separated into four groups (Table 1). Groups of fish were randomly assigned to tanks and fish were identified through their individual PIT tag number for the duration of the experiment. All four tanks, two stocking tanks and two holding tanks, received the same water supply through a splitter and had the same flow rates. Fish were initially held in the stocking tanks and for all tests, including baseline reflex testing and air exposures, fish were returned to two separate holding tanks. Water temperatures ranged from 24.5 to 29°C, depending on the time of day, and dissolved oxygen within the tanks ranged from 6.5 to 7.5 mg/L.

**Table 1:** Numbers of fish within each group and total numbers of fish tested at each exposure

Group	Air exposure (min)						Total
	0	2.5	3	4	5	8	
I	7				2	3	12
II	3	2	2		1		8
III	4		4				8
IV		3		3			6
Total	14	5	6	3	3	3	34

### Reflex and stress testing

Reflexes that were consistently present in the unstressed, control fish were identified. The reflexes tested in restrained fish included vestibular–ocular response (VOR), head complex, mouth reflex and body flexion. Reflexes tested in unrestrained fish were tested in the water and consisted of equilibrium and the tail grab. Reflexes were scored as either present (0) or absent (1), not on the strength of the reflex response. If there was uncertainty that the reflex was present, it was scored as absent. VOR was noted as present when the fish was rotated laterally and the eye rotated in the socket and remained fixed on the investigator. Head complex was present when the fish exhibited rhythmic movements of mouth gape and operculum flare for 5 s. The mouth reflex was present if the mouth returned to the closed position after being opened with a probe. The reflex was present for body flexion if the fish flexed on a flat surface. For the equilibrium reflex, fish were placed in the tank upside down and if the fish returned to an upright position, the equilibrium reflex was scored as present. Once upright, the investigators lightly grasped the caudal fin and if the fish attempted to burst-swim away, the reflex was marked as present.

Once reflexes were identified in unstressed fish, they were exposed to different air exposure treatments beginning at 2.5-min and up to 8 min (Table 1). All fish in the study had reflexes tested twice, once under unstressed conditions and once after a single air exposure. Individual fish were removed from the stocking tanks and placed in a foam-lined container for the duration of the air exposure. PIT tag numbers and signs of infection were recorded during the air exposure. At the completion of the air exposure in the foam-lined container, reflexes were tested in the following order: body flexion, mouth reflex, head complex and VOR. Fish were then returned to holding tanks and the equilibrium reflex was tested followed by the tail grab. Fish were then monitored for 7 days for delayed mortality. The process of assessment of reflexes took an average of 15 s, excluding the equilibrium reflex.

The proportion of reflex impairment for individual fish was calculated as the total reflex impairment by the total number of tested reflexes. Delayed mortality was scored as survival (0) or delayed mortality (1). Fish that survived one week from the stressor were scored as surviving. To obtain an LD_50_ for the four groups of fish, the group averages of reflex impairment proportion and delayed mortality were also calculated. The proportional contribution of each reflex towards total reflex impairment was calculated as the average of each impaired reflex divided by the sum of all impaired reflex averages (Davis, 2010).

### Blood sampling

Blood was drawn from a sub-sample of fish prior to reflex testing via caudal puncture with a heparinized syringe. Whole blood measurements were performed using the VetScan i-STAT 1 (Abaxis, Union City, CA, USA) with the i-STAT CG4+ and i-STAT CG8+ cartridges. CG4+ cartridges measured: pH, pCO_2_, pO_2,_ BE_ecf_ (base excess in extracellular fluid), HCO_3_, TCO_2_ (total carbon dioxide), sO_2_ (oxygen saturation) and lactate concentrations. CG8+ cartridges measured: pH, pCO_2_, pO_2,_ BE_ecf_, HCO_3_, TCO_2_, sO_2_, Na^+^, K^+^, iCa (intracellular calcium), glucose, haematocrit and haemoglobin. Cartridges were stored in the dark in their original packaging at 2°C. Before testing, the cartridges were allowed to equilibrate to the ambient temperature of 28°C. Whole blood was first measured with the CG4+, followed by the CG8+. Blood gases and pH measurements were taken from the CG4+ cartridges and values obtained from the CG8+ cartridges were discarded. The remainder of the blood samples were centrifuged and the resulting plasma was frozen and stored at −20°C.

### Laboratory testing

Frozen plasma was thawed at the University of Miami and lactate and glucose assays run. The concentration of lactate and glucose were measured using commercial assay kits (Sigma-Aldrich Co.).

### Statistical analysis

The proportion of reflex impairment for each fish in response to air exposure were fitted to a linear regression model and individual fish’s mortality were fitted to a binomial generalized linear model with a logit link. The relationship between air exposure and average reflex impairment in groups was calculated in a generalized linear model as was the relationship between reflex impairment and mortality in groups. Relationship were tested for significance with *F =* MS_regression_/MS_residual_ using RStudio and the MASS package. Significance was set at *P* = 0.05 for all tests. The median lethal dose (LD_50_) of reflexes for individual fish and groups were identified from the coefficients of the fitted models. The confidence intervals for the LD_50_ were obtained through bootstrapping via the boot package (Canty and Ripley, 2016).

The correlations between reflex impairment, air exposure, delayed mortality and whole blood physiological parameters determined by the i-STAT were measured using Pearson correlation with a significance levels of *P* = 0.05. Variables that were significantly correlated were included in two generalized linear models, one to predict delayed mortality and the other to predict reflex impairment. For individual fish, a binomial GLM model was used with a logit link to predict mortality. Significant variables were identified with stepwise regression by AIC using the MASS package in R (Venables and Ripley, 2002).

Measurements taken by the i-STAT were compared to results obtained from the traditional laboratory assays of lactate and glucose concentrations as well as for Na^+^ and K^+^ using regression analysis. All data analysis was completed through RStudio (RStudio Team, 2015) and R programs (R Development Core Team, 2008).

## Results

### Reflex and stress testing

**Figure 1:**
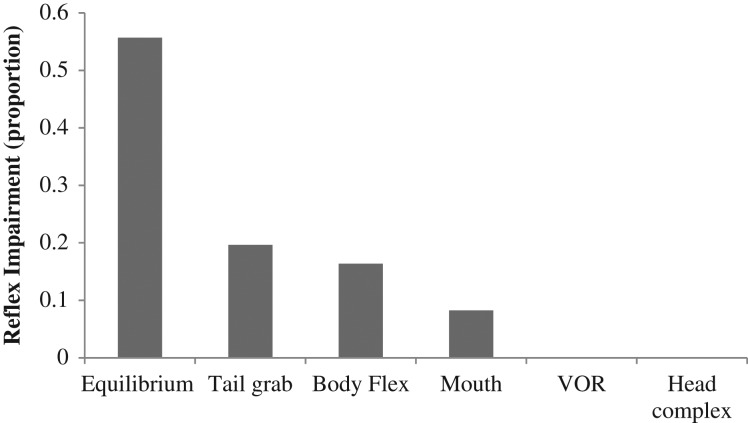
Contribution of each reflex action to impairment proportion.

The reflexes most frequently impaired in yellowtail snapper were equilibrium, followed by the tail grab reflex (Fig. 1). The VOR and the head complex reflex were always present in air-exposed fish.

**Figure 2:**
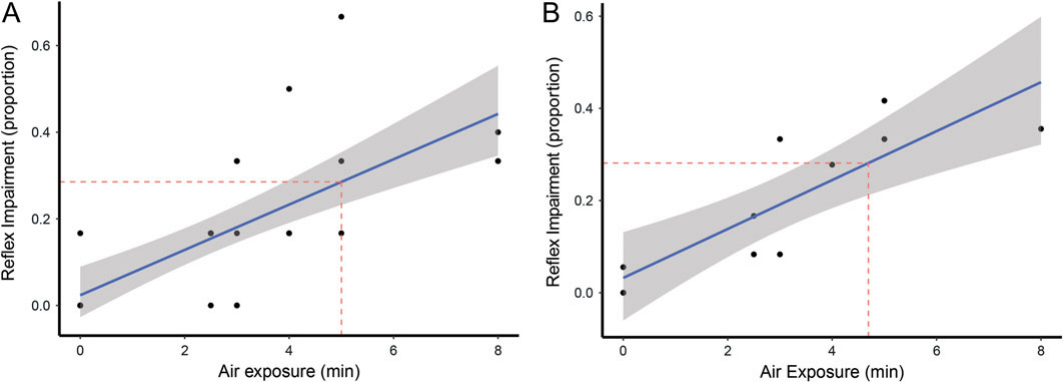
(**A**) Linear regression model fitted to air exposure (min) and reflex impairment (proportion). Points are individual fish impaired reflex scores at given air exposures. (*P* < 0.001, *n* = 32). Gray area around fitted line represents 95% confidence interval. Dashed red line represents the median lethal dose of reflex impairment and the corresponding air exposure. (**B**) Linear regression model fitted to air exposure (min) and reflex impairment (proportion). Points are group averages of impaired reflex scores at given air exposures. (*P* < 0.001, *n* = 11). Gray area around fitted line represents 95% confidence interval. Dashed red line represents the median lethal dose of reflex impairment and the corresponding air exposure.

The relationship between air exposure and reflex impairment for individual fish was significant (*P* < 0.001, *n* = 32; and Fig. 2A). At 4.3 min, 25% of the reflexes were impaired in the tested fish. The air exposures only extended to 8 min, however, if the model correctly predicted reflex impairment in response to air exposures, 50% of reflexes would be impaired at 9 min of air exposure. The results from individual fish are comparable to the results obtained from group averages. The group averages were fitted to a generalized linear model, rather than a linear model, however, 25% of the reflexes were impaired at 4.1 min of air exposure (Fig. 2B). The relationship between air exposure and average reflex impairment was significant (*P* < 0.001, *n* = 11; Table 2).

**Table 2:** Model results of air exposure on reflex impairment in individual fish (linear model) and group averages (generalized linear model)

	DF	SS	MSS	*F* value	Pr(>*F*)
Response: reflex impairment (individuals)					
Air	1.00	0.57	0.57	37.64	<0.001
Residuals	30.00	0.45	0.02		
Coefficients:	Est.	Std. error	*t* value	Pr(>|*t*|)	
(Intercept)	0.02	0.03	0.81	0.42	
Air	0.05	0.01	6.14	<0.001	
Adj. *R*^2^	0.56				
Response: reflex impairment (groups)					
Air	1	0.17	0.17	22.96	<0.001
Residuals	9	0.07	0.01		
Coefficients:	Est.	Std. error	*t* value	Pr(>|*t*|)	
(Intercept)	0.03	0.04	0.76	0.47	
Air	0.05	0.01	4.79	<0.001	
Adj. *R*^2^	0.69				

**Figure 3:**
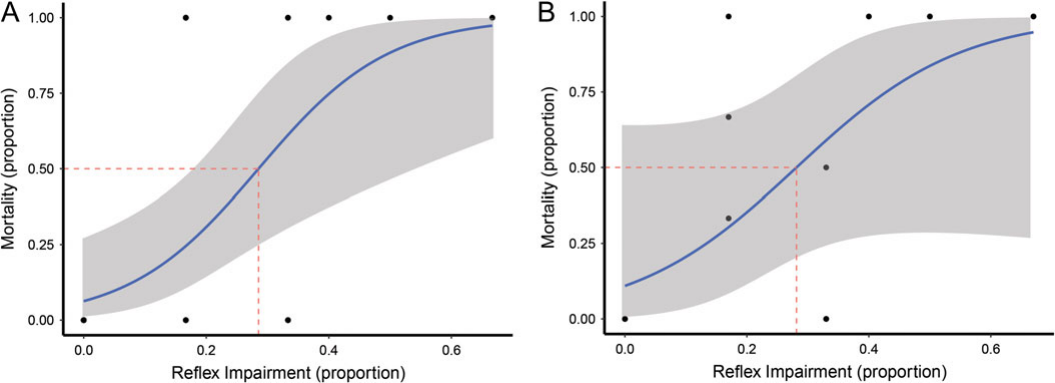
(**A**) Binomial model fitted to reflex impairment (proportion) and mortality (proportion). Points are individual fish reflex impairment and corresponding mortality (*P* < 0.001, *n* = 30). Gray area around fitted line represents 95% confidence interval. Dashed red line represents the median lethal dose of reflex impairment. (**B**) Generalized linear regression model fitted to average reflex impairment (proportion) for each group and corresponding average group mortality (*P* = 0.03, *n* = 12). Gray area around fitted line represents 95% confidence interval. Dashed red line represents the median lethal dose of reflex impairment.

**Figure 4:**
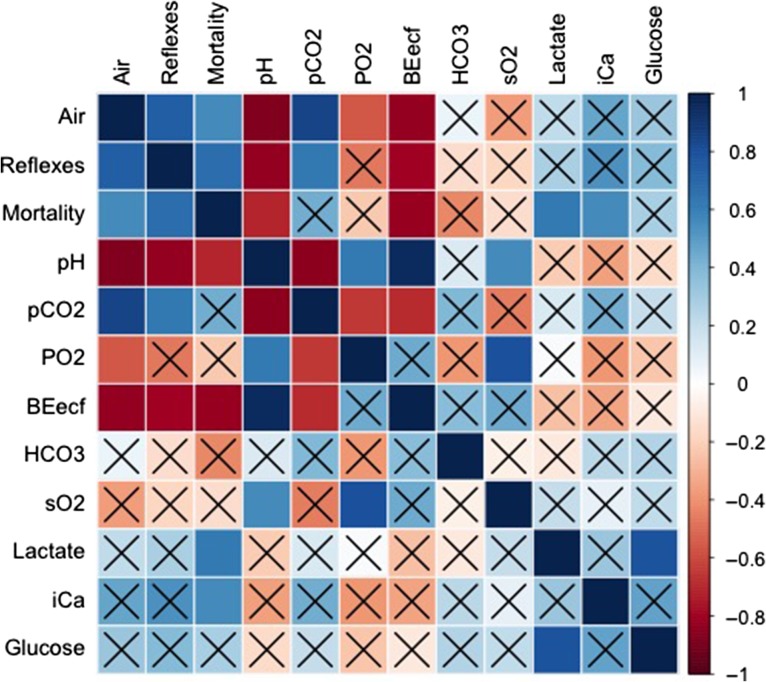
Results of Pearson correlation between air exposure, reflex impairment, physiological parameters and delayed mortality. Variables that are not significant (*P* > 0.05) are crossed out.

The relationship between reflex impairment and delayed mortality for individual fish was significant, (*P* < 0.001, *n* = 32, Fig. 3A), as was the average mortality given average reflex impairment within a group (*P* = 0.03, *n* = 12; Table 3 and Fig. 3B). The LD_50_ of reflex impairment for individual fish occurred with 29% of reflexes impaired (95% CI: [0.15, 0.42], *n* = 31), which corresponds to an air exposure of 5 min (Figs 3 and 4). The LD_50_ of reflex impairment for groups was 28% of reflexes impaired (95% CI: [−0.20, 0.76], *n* = 12), occurring at 4.7 min of air exposure (Figs 2 and 3).

**Table 3:** Model results of proportion of reflex impairment on delayed mortality in individual (binomial model) fish and group averages (generalized linear model)

	Est.	Std. error	*z*-value	Pr(>|*z*|)	
Response: delayed mortality (individual)					
(Intercept)	−2.70	0.87	−3.10	<0.001	
Reflex Impairment	9.47	3.40	2.79	<0.001	
	DF	Dev	Resid. DF	Resid. dev.	Pr(>Chi)
Null			31	38.02	
Reflex impairment	1	12.60	30	25.42	<0.0001
	Estimate	Std. error	*t*-value	Pr(>|*t*|)	
Response: delayed mortality (group)					
(Intercept)	0.11	0.17	0.62	0.55	
Reflex impairment	1.37	0.53	2.59	0.03	
	DF	Dev	Resid. DF	Resid. dev.	Pr(>Chi)
Null			11	2.28	
Reflex impairment	1	0.92	10	1.37	<0.01

### i-STAT measurements

**Figure 5:**
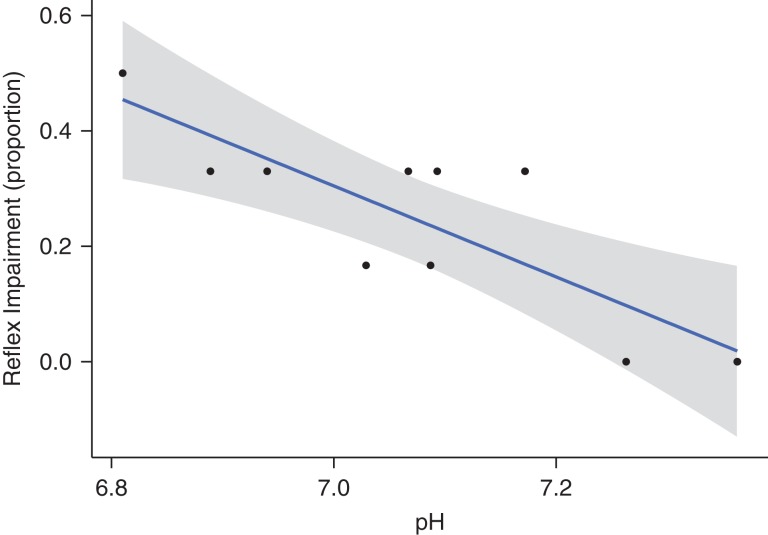
Linear regression model fitted to pH levels and reflex impairment and 95% confidence interval (gray area).

Blood was drawn from 12 fish and successfully tested with i-STAT cartridges (Table 4). Within this sub-sample of fish, air exposure, pH and BE_ecf_ were significantly correlated to reflex impairment. Delayed mortality was significantly correlated to air exposure, and changes in pH, BE_ecf_ and lactate concentration (Fig. 4). While the linear regression model to predict reflex impairment was significant for air exposure, the model selected through the AIC stepwise regression retained only pH as a significant predictor (Table 5 and Fig. 5). The linear regression model to predict delayed mortality had BE_ecf_ and lactate as significant predictors (Table 6). Blood sampling did not have a significant effect on mortality (Table 7).

**Table 4:** Mean values reported by CG4+ and CG8+ cartridges

i-STAT parameter	Unit	Mean	SEM
CG4+ cartridge			
pH	mmHg	0.25	0.04
pCO_2_	mmHg	0.36	0.15
PO_2_	mmol/L	7.08	0.05
BE_ecf_	mmol/L	16.60	1.60
HCO_3_	mmol/L	49.73	18.09
TCO_2_	mmol/L	−25.36	0.79
sO_2_	%	4.67	0.28
Lac	mmol/L	5.71	0.36
CG8+ cartridge			
Na	mmol/L	6.00	0.26
K	mmol/L	55.44	9.15
iCa	mmol/L	179.00	–
Glu	mg/dL	6.00	0.40
Hct	%PVC	1.69	0.03
Hb	g/dL	53.18	5.63

**Table 5:** Summary of results from linear regression model predicting reflex impairment using i-STAT parameters and air exposure. Model 1 is the complete model and Model 2 is preferred by the AIC

	DF	SS	MSS	*F* value	Pr(>F)
Model 1—response: reflex impairment					
Air	1	0.13	0.13	9.62	<0.05
pH	1	0.03	0.03	2.44	0.18
pCO_2_	1	0.01	0.01	0.40	0.55
BE_ecf_	1	0.01	0.01	0.39	0.56
Residuals	5	0.07	0.01		
	Estimate	Std. error	*t* value	Pr(>|*t*|)	
Coefficients:					
(Intercept)	14.23	13.01	1.09	0.32	
Air	0.03	0.05	0.59	0.58	
pH	−1.78	1.55	−1.15	0.30	
pCO_2_	−0.02	0.02	−0.89	0.42	
BE_ecf_	0.05	0.07	0.63	0.56	
Adj. *R*^2^	0.50				
Model 2—response: reflex impairment					
pH	1	0.16	0.16	16.58	<0.01
Residuals	8	0.08	0.01		
	Est.	Std. error	*t* value	Pr(>|*t*|)	
Coefficients:					
(Intercept)	5.82	1.37	4.25	<0.05	
pH	−0.79	0.19	−4.07	<0.05	
Adj. *R*^2^	0.63

**Table 6:** Summary of results from linear regression model predicting delayed mortality using i-STAT parameters, reflex impairment and air exposure.

	DF	SS	MSS	*F* value	Pr(>*F*)
Model 1—response: delayed mortality					
Reflex impairment	1	1.06	1.06	16.38	0.02
Air	1	0.02	0.02	0.34	0.59
pH	1	0.23	0.23	3.58	0.13
BE_ecf_	1	0.46	0.46	7.11	0.06
Lactate	1	0.37	0.37	5.80	0.07
Residuals	4	0.26	0.06		
	Est.	Std. error	*t* value	Pr(>|*t*|)	
(Intercept)	−15.35	17.44	−0.88	0.43	
Reflex impairment	0.37	0.94	0.40	0.71	
Air	−0.08	0.10	−0.82	0.46	
pH	1.35	2.12	0.64	0.56	
BE_ecf_	−0.24	0.11	−2.24	0.09	
Lactate	0.09	0.04	2.41	0.07	
Adj. *R*^2^	0.76				
Model 2—response: delayed mortality					
BE_ecf_	1	1.58	1.58	28.19	<0.001
Lactate	1	0.43	0.43	7.60	<0.05
Residuals	7	0.39	0.06		
	Est.	Std. error	*t*value	Pr(>|*t*|)	
Coefficients:					
(Intercept)	−3.30	0.73	−4.50	<0.001	
BE_ecf_	−0.13	0.03	−4.45	<0.001	
Lactate	0.09	0.03	2.76	<0.05	
Adj. *R*^2^	0.79

**Table 7:** Results of binomial GLM predicting mortality given air exposure, reflex impairment or blood sampling

	Estimate	Std. error	*t* value	Pr(>|*t*|)
Coefficients:				
(Intercept)	0.06	0.11	0.61	0.55
Reflex impairment	1.42	0.57	2.52	<0.05
Blood	−0.05	0.14	−0.39	0.70
Air	0.01	0.04	0.34	0.74

### i-Stat validation

**Figure 6:**
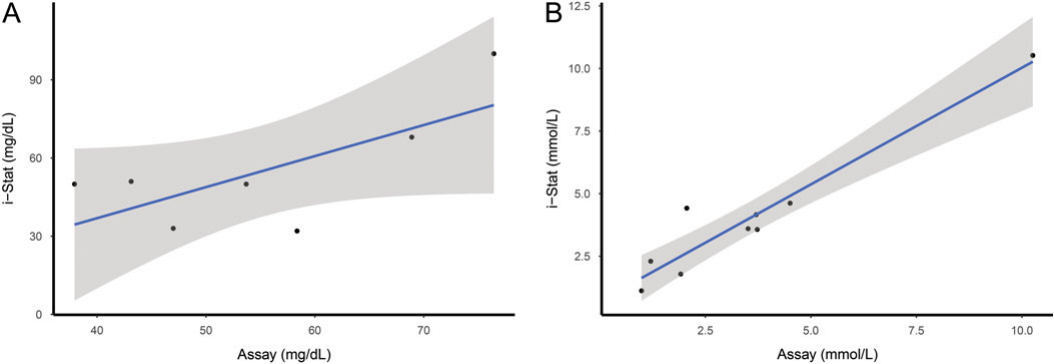
(**A**) Linear regression model of glucose concentrations measured with assay and by i-STAT CG8+. Gray area around fitted line represents 95% confidence interval. (**B**) Linear regression model of lactate concentrations measured with assay and by i-STAT CG8+. Gray area around fitted line represents 95% confidence interval.

Lactate and glucose concentrations were successfully tested in the laboratory using 7 of the 12 blood-sampled fish. Insufficient whole blood remained after two i-STAT cartridge tests to provide adequate plasma for sampling. The linear regression model for the relationship between the values obtained from the commercial glucose assay compared to the i-STAT measurements was not significant (*P* = 0.07, *n* = 7) and the 95% confidence interval was not well fitted to the data (Fig. 6). However, the linear regression model for the relationship between the values obtained from the commercial lactate assay and the i-STAT measurements was significant (*P* < 0.001, *n* = 7) and the 95% confidence interval closely fitted to the regression line (Fig. 6). The commercial assay kit values obtained for lactate were not significant predictors for reflex impairment or delayed mortality with the reduced sample size (*P* = 0.17, *n* = 7; *P* = 0.88).

## Discussion

The RAMP method in this study provided a useful indicator of release mortality in yellowtail snapper with respect to air exposure. Additionally, reflex impairment showed strong relationships to physiological parameters, particularly those relating to acid–base balance in teleosts. This has the potential for estimating release mortality within the fishery.

The most commonly impaired reflex, equilibrium and tail grab, were the two reflexes observed once the fish were placed back in the water. These reflexes play an important role in predator evasion and the ability of the fish to return to its natural position above the reef complex (Ryer *et al.*, 2004; Diamond and Campbell, 2009; Davis, 2010; Gilman *et al.*, 2013). The equilibrium response is perhaps the easiest to observe by recreational anglers as it is naturally witnessed when fish are returned to the water after de-hooking (Brownscombe *et al.*, 2013). In contrast to reflex impairments observed in other teleost species, the VOR and the head complex reflex were never impaired (Davis, 2010, 2007). Body flexion was the most often impaired reflex in rock sole while body flexion and the VOR were most impaired in halibut species (Davis, 2007). This highlights the necessity of identifying appropriate reflexes in the species of interest, in both unstressed and stressed fish, as different species may have vastly different patterns in which reflexes become most frequently impaired in response to stress. These reflexes are components of complex volitional behaviours that may also become impaired in response to increasing stress; each species’ unique morphology and physiology will dictate how stress affects the complex behaviours necessary for survival (Davis, 2010, 2005).

Reflex impairment was significantly related to air exposure in both individual fish and when fish were averaged over groups. The point at which reflex impairment was 50% (9 min) was not observed in terms of the experiment, but rather represents a number obtained from the model. If this estimate holds true, and the model is extended into longer time scales of air exposure, that length of air exposure is outside the reasonable period these fish could be exposed to air before being returned to the water without detriment. The LD_50_ for proportion of reflex impairment were 0.29 and 0.28 for individuals and averages, respectively, corresponded to air exposures approaching 5 min. This length of air exposure is most likely beyond the time fish would be out of the water if caught and de-hooked by an experienced angler. However, with inexperienced, recreational anglers this length of air exposure may be observed before the fish is returned to the water (Meka, 2004). Additionally, if the fish are gut hooked or with multiple hooks, the time to de-hook would most likely increase, potentially decreasing post-release survival rates (Rummer, 2007; Bartholomew *et al.*, 2008).

The LD_50_ of proportion of reflex impairment in individual yellowtail snapper calculated in the present study is relatively low compared to other species in which reflex impairment has been assessed. Species with a higher inflection point (LD_50_) could be viewed as more resilient to stress. Halibut (*Hippoglossus stenolepis*) experienced 50% mortality near reflex impairment of 0.8, while rock sole (*Lepidopsetta polyxystra*) was ~0.4 (Davis and Ottmar, 2006). The stressors used to assess reflex impairment and mortality were slightly different for these two species, rock sole were towed prior to reflex assessment while halibut were towed and then exposed to air before reflex assessments. However, the reflex impairment LD_50_ in yellowtail snapper is quite similar to pollock (*Theragra chalcogramma*) (0.2) and salmon (*Oncorhynchus kisutch*) (0.1), which were only exposed to towing and not exposed to the air (Davis, 2007). Other studies utilizing impaired reflex methodology found that air exposure was the most significant predictor of delayed mortality; however, it was often used in combination of other stressors, such as tow duration as was the case with halibut (Davis and Ottmar, 2006). The morphological and physiological variations of all species as well as the stressors present in each unique fishery will dictate how susceptible each species is to the stress of being caught. Furthermore, the use of reflex impairment to predict delayed mortality in more sensitive species can be challenging due to high rates of mortality after comparatively small impairments in reflexes.

Differences between conditions in the controlled environment of this study and those present in the fishery may impact the relationship between reflex impairment and delayed mortality. For example, the laboratory conditions under which the fish were held do not adequately mimic the natural environment fish are discarded into after being caught, such as habitat cover, social interactions and predation. Most likely, these differences will negatively impact reflex impairment and thus increase the likelihood of delayed mortality. Behaviours and reflexes necessary to avoid predation and return to a suitable habitat on the reef could potentially be greatly diminished resulting from the air exposure prior to discard. Loss of equilibrium upon return to the water has the potential to make discarded fish vulnerable to predation, as does the inability to burst swim away from a stimulus (Cooke and Philipp, 2004; Danylchuk *et al.*, 2007; Brownscombe *et al.*, 2013).

The i-STAT and other portable POC devices have begun to gain more attention for use in field and fishery settings (Gallagher *et al.*, 2010; Harter *et al.*, 2014; Stoot *et al.*, 2014). However, these devices were originally intended for use in a clinical setting; the measurements and calculations of blood parameters are based on algorithms written for mammalian blood. Teleost red blood cells are nucleated, unlike mammalian red blood cells, which can cause some teleost species to have strong Bohr/Haldane and/or Root effects (Barton and Iwama, 1991; Wendelaar Bonga, 1997; Wells and Dunphy, 2009). These can introduce bias in the measurements of blood gases as well as acid–base interactions (Harter *et al.*, 2014). The Root effect can limit the amount of oxygen bound to the haemoglobin and in turn can affect acid–base regulation. The blood gas values from the i-STAT in this study were not significant for predicting delayed mortality or reflex impairment, however, base excess (BE_ecf_), pH and lactate were significant factors in reflex impairment and survival. While the actual values measured have the potential to be influenced by the differences in fish red blood cells and would not be used predict survival, this study focused on these parameters in relation to larger, whole animal responses to stress.

The i-STAT measured components of both of these pathways: the respiratory pathway through pCO_2_ and the metabolic pathway *via* HCO_3_ and BE_ecf_, in addition to overall acid–base status as pH. The respiratory parameter measured by the i-STAT (pCO_2_) was not significantly correlated with reflex impairment or delayed mortality; however, both BE_ecf_, which was negative in yellowtail snapper indicating a base deficit in the blood and a metabolic acidosis, and the reduction in pH, were significantly correlated. Base excess and pH were also negatively correlated with air exposure. BE_ecf_ measurements with the i-STAT were calculated from HCO_3_ and pH levels. These calculations were based on 37°C and the amount of base needed to return plasma pH to 7.4 (BE_ecf_ = HCO_3_− 24.8 + 16.2 (pH-7.4); i-STAT Technical Bulletin, 2013). There are two confounding factors with the i-STAT BE_ecf_ measurement. First, as fish are ectotherms, the temperature of yellowtail blood in this study ranged from 24.5 to 29°C, which differs from mammalian body temperature of 37°C. Second, the i-STAT may not be able to detect the shifts in the levels of HCO_3_ in teleosts as a result of the cartridges calibrated for mammalian blood.

Consistent with air exposure and acidosis, an increase in blood lactate levels was observed. A build-up of lactate occurs when the animal receives too little, or in the case of the present study, no oxygen from the air exposure, resulting in impaired cellular respiration. This in turn forces cell to metabolize glucose anaerobically, resulting in the formation of lactate (Butler *et al.*, 1979; Hobbs *et al.*, 2010; Holeton and Randall, 1967). Impaired cellular respiration leads to lactic acidosis and a decrease in pH values, which was observed to be significant for predicting reflex impairment (Hobbs *et al.*, 2010). The reduction of cellular O_2_ reduces the amount of ATP available to the muscles of the fish (Wu, 2002), which could be the mechanism causing the impairment of reflexes observed in this study. While elevations in lactate, a secondary response to acute stress in teleosts, is elevated in response to handling or capture stresses (Schwalme and Mackay, 1985; Wendelaar Bonga, 1997; Barton, 2002); it is not often a good predictor of mortality (Davis, 2001; Skomal, 2007). However, in the present study, delayed mortality was significantly predicted though increased concentration of lactate.

## Conclusions

This study demonstrated that length of air exposure is a significant predictor for reflex impairment in yellowtail snapper. With fish that were not blood sampled, reflex impairment was a significant predictor for delayed mortality. With blood-sampled fish, pH was a better predictor for the proportion of reflex impairment than air exposure. In addition, lactate and BE_ecf_ concentrations predicted delayed mortality better than reflex impairment. In the absence of blood physiology parameters, the use of reflex impairment is an appropriate method to assess the rates of the post-release survival in field conditions. The i-STAT lactate measurements may be a valid method to predict delayed mortality and the lactate and glucose measurements were well correlated to values obtained in the laboratory.

There is scant information on rates of discard mortality in undersized yellowtail snapper. The 2003 assessment of yellowtail snapper set the discard mortality rate at 30% while the 2012 assessment set the rate at 10% and included sensitivity runs using discard mortality rates up to 30% (O’Hop *et al.*, 2012). This uncertainty combined with the high levels of yellowtail snapper discarded within Florida waters (Bohnsack, 2000) highlights the necessity of studies like this to understand the full impacts of the fishery.

## Funding

This work was funded through the International Seafood Sustainability Foundation, the Cooperative Institute of Marine and Atmospheric Science (NOAA Cooperative Agreement NA10OAR4320143) and the Rosenstiel School of Marine and Atmospheric Science Mary Roche Fellowship. Animal care and use were conducted per IACUC protocols (UM IACUC 15-150).
